# Lectin galactoside-binding soluble 3 binding protein mediates methotrexate resistance in choriocarcinoma cell lines

**DOI:** 10.1080/21655979.2021.2022844

**Published:** 2022-01-17

**Authors:** XiaoJing Chen, Yite Xue, Lingfang Wang, Yang Weng, Sen Li, Weiguo Lü, Xing Xie, Xiaodong Cheng

**Affiliations:** a Key Laboratory of Women’s Reproductive Health of Zhejiang Province, Women’s Hospital, School of Medicine, Zhejiang University, Hangzhou, China; b Department of Gynecologic Oncology, Women’s Hospital, School of Medicine, Zhejiang University, Hangzhou, China

**Keywords:** Lectin galactoside-binding soluble 3 binding protein (LGALS3BP), choriocarcinoma, methotrexate resistance, RNA–sequencing

## Abstract

Choriocarcinoma is one of the most aggressive gestational trophoblastic neoplasias (GTN). Methotrexate (MTX) resistance is the main cause of treatment failure in choriocarcinoma. However, the mechanism of MTX resistance in choriocarcinoma is poorly known. This study aims to explore the function of Lectin galactoside-binding soluble 3 binding protein (LGALS3BP) in MTX-resistance in choriocarcinoma cells. Gradual dose escalation of MTX was used to establish MTX-resistant choriocarcinoma cells (JAR-MTX and JEG3-MTX cell lines). RNA-sequencing was used to explore the differentially expressed genes. Plasmids or SiRNA transfection was used to regulate the expression of LGALS3BP. ELISA was used to detect the concentrations of LGALS3BP in the serum of MTX-sensitive and MTX-resistant patients. qRT-PCR, Western blot, and CCK-8 assay were used to determine the effects of LGALS3BP on MTX-resistance in JAR and JEG3 cells. The results showed the relative resistance index (RI) of MTX is 791.50 and 1040.04 in JAR-MTX and JEG3-MTX, respectively. LGALS3BP was up-regulated in MTX-resistant cells compared to original cells in both RNA and protein level. The concentrations of LGALS3BP were higher in the sera of MTX-resistant patients than in MTX-sensitive patients. Knocking down LGALS3BP can reverse the MTX-resistance in JAR-MTX and JEG3-MTX cells. In summary, we preliminarily established two MTX-resistant cells, and performed RNA-sequencing, and found LGALS3BP may play important role in MTX-resistance. Our work not only provides a research tool (MTX-resistant cells) for other researchers, but gives some hint on how MTX resistance is regulated.

## 1. Introduction

Gestational trophoblastic neoplasia (GTN) is a group of pregnancy-related malignant disorders, including invasive moles, choriocarcinoma, placental-site trophoblastic tumors, and epithelioid trophoblastic tumors [1–3]. Among these, Choriocarcinoma is the most aggressive and can metastasize to distant organs, with a poor prognosis overall [4,5]. Chemotherapy is the main treatment of choriocarcinoma. Although the currently used therapies including methotrexate (MTX), actinomycin-D (ActD), and other chemotherapeutics result in acceptable remission rates [3,6], relapse and development of drug resistance remain a challenge in the clinic [7–9]. It is reported that 25% low-risk and 10 ~ 20% high-risk GTN patients failed to achieve complete remission after primary chemotherapy [7,8], Chemotherapeutic resistance has been the most important cause of treatment failure in choriocarcinoma. However, the underlying mechanisms of drug resistance in GTN are poorly understood. Methotrexate (MTX), an antifolate that can suppress dihydrofolate reductase in DNA synthesis [10] has been widely used as the first-line treatment of choriocarcinoma. In addition, MTX is also used to treat high-risk GTNs when combing with other chemotherapy regimen such as etoposide, MTX, ActD/cyclophosphamide plus vincristine (EMA/EO regimens) [6,8]. With serious acquired drug resistance, more than 30% of patients develop drug-resistance for MTX mono-therapy. It is important to unveil the mechanism of MTX-resistance in choriocarcinoma in order to improve its prognosis. As a result, establishing MTX-resistance choriocarcinoma cell line is of great significance in exploring the mechanism of MTX-resistance. Lectin galactoside-binding soluble 3 binding protein (LGALS3BP), also known as 90 K or Mac-2 BP, is over-expressed in many cancers, such as prostate, colorectal and lung cancer [11–13], and has been reported to play important role in tumor metastatic processes, and is associated with low survival rate in human cancers [12]. Mechanically, on the one hand, as a secretory glycoprotein, LGALS3BP interacts with extracellular matrix elements and members of the galectin family, facilitating cell adhesion to matrix proteins by interaction with β1 integrin subunits which play important role in cancer metastasis. On the other hand, binding of LGALS3BP to integrin on tumor cells activates the Akt and Raf-Erk pathways, which was associated with increased survival, proliferation, motility, and migration of cancer cell lines [14]. However, there is limited research exploring its function in drug resistance. It is reported LGALS3BP can induce 17-N-Allylamino-17-demethoxygeldanamycin resistance in lung cancer cell Line, but the detailed mechanism is unknown [14]. Further study is needed to study the function and mechanism of LGALS3BP in MTX-resistance in choriocarcinoma.

This study focused on the relationship between LGALS3BP expression and MTX-resistance in choriocarcinoma to verify whether LGALS3BP can regulate the sensitivity of choriocarcinoma cell lines toward MTX. In order to uncover the mechanisms involved in MTX-resistance in choriocarcinoma and the effect of LGALS3BP on the effectiveness of MTX treatment, we established MTX-resistant choriocarcinoma cells by gradual dose escalation of MTX, and found LGALS3BP was up-regulated in MTX-resistant cells compared to original cells. Meanwhile, we found that the sera concentration of LGALS3BP was higher in MTX-resistant patients than MTX-sensitive patients. Moreover, knocking down of LGALS3BP can significantly reverse the resistance of MTX while overexpressing of LGALS3BP will lead to MTX-resistance in choriocarcinoma.

## 2. Materials and methods

### 2.1. Cell culture and establishment of MTX-resistant cell lines

Two human choriocarcinoma cancer cell lines, JAR and JEG3, were obtained from the American Type Culture Collection (ATCC, Manassas USA), maintained in Women’s Hospital, School of Medicine, Zhejiang University, and authenticated and tested for Mycoplasma contamination. Cells used in this study were not passaged continuously for more than 3 months. JAR cells were cultured in RPMI-1640 medium (Gibco, USA), and JEG3 cells were cultured in EBSS-MEM medium (Gibco, USA). All cell lines were supplemented with 10% fetal bovine serum (FBS) (Gibco, USA), maintained at 37°C in 5% CO^2^ and detached from the culture plates using trypsin/EDTA solution (Gino biotech, Shanghai, China) [9]. MTX was purchased from Selleck Chemicals. To establish the MTX-resistant cell lines (JAR-MTX and JEG3-MTX), firstly JAR and JEG3 cells according to the concentration of 50% inhibition (IC_50_) of MTX, the IC_50_ of MTX was 0.44 μM that as the base concentration, then gradually increasing dose of MTX. When the concentration of the drug was increasing, the cells cultured for 24 h, then washed with PBS (Gibco, USA) for 3–5 times, and cultured in medium without MTX. Surviving cells were collected and the above protocol was repeated 3–5 times, until the cells could grow stably in 88 μM MTX [15].

### 2.2. Observation of cytological morphology

To compare the cell morphology of MTX-resistant JAR and JEG3 cells, with that of original JAR and JEG3 cells, cells in the logarithmic phase were replaced with fresh culture medium and photographed with an inverted microscope.

### 2.3. Cell proliferation assay

Cell proliferation was measured by CCK8 kit (DOJINDO, Japan). Briefly, cells were seeded 5 × 10^3^ per well into 96-well plates. After 24, 48, 72 and 96 hours of culture, a 10 μL CCK8 solution was added to each well and incubated for 1 hour. The absorbance at 490 nm was measured with a Varioskan Flash microplate reader (Thermo Fish Scientific, Waltham, MA, USA) [16].

### 2.4. Cell viability assay

Cells were seeded into 96-well plates at a density of 5 × 10^3^ cells per well. After one day of growth, the cells were treated with different concentrations of Etoposid (VP – 16) (Selleck, USA) 5-Fluorouracil (5 – FU) (Selleck, USA), Methotrexate (MTX) (Selleck, USA), Paclitaxel (TAXOL) (Selleck, USA), or ActD (Selleck, USA) and incubated at 37°C for 72 hours. Cell viability was detected by CCK – 8 viability assay at 72 hours after drug treatment [9]. The absorbance at 490 nm was measured with a Varioskan Flash microplate reader (Thermo Fish Scientific, Waltham, MA, USA) and the results were calculated using SPSS software.

The concentrations of drugs used in MTX-resistant cells were listed below:

VP-16:0.05, 0.1, 0.2, 0.5, 1, 2, 5, 10, 20 ng/ml;

5-FU:0.05, 0.1, 0.5, 1, 5, 10, 20, 50 μg/ml;

TAXOL:0.06, 0.1, 0.3, 0.6, 1, 3, 6, 10, 30 nM;

MTX:1, 4, 10, 40, 100, 400, 1000 μM;

ActD:0.1, 0.4, 0.8, 1, 4, 8, 10, 40 μg/ml.

The concentrations of drugs used in original cells were listed below:

VP-16:0.005, 0.01, 0.05, 0.1, 0.5, 1, 2 ng/ml;

5-FU:0.05, 0.1, 0.5, 1, 5, 10, 20 μg/ml;

TAXOL:0.06, 0.1, 0.3, 0.6, 1, 3, 6, 10, 30 nM;

MTX:0.01, 0.04, 0.1, 0.4, 1, 4, 10, 40 μM;

ActD:0.1, 0.4, 0.8, 1, 4, 8, 10, 40 μg/ml.

(Note: It is converted into a uniform concentration unit for subsequent statistics)

### 2.5. Cell cycle analysis

Cell cycle analysis was performed using Propidium Iodide Stain (PI) (MultiSciences, China) following manufacturer’s instructions. Briefly, cells were harvested, washed with PBS, and resuspended in binding buffer at a concentration of 1 × 10^6^ cells/ml. 100 μL cell suspension of each sample was fixed in ice-cold 70% ethanol overnight and incubated with 50 μg/ml PI at 4°C for 30 minutes [17]. The stained cells were then run on a Beckton Dickinson FACScan (BD Biosciences, USA) flow cytometer.

### 2.6. RNA isolation and qRT-PCR

Total RNA was isolated using an RNA extraction kit (TaKaRa, Japan) and 1000 ng RNA was used to reverse-transcribed into cDNA by the reverse transcription cDNA kit (TaKaRa, Japan). Quantitative real-time PCR was performed with 20 ng of cDNA using the SYBR® Premix ExTaq™ (TaKaRa, Japan). The mix was added to 96-well plate detected by a Biosystems 7900HT fast real-time PCR system (Life Technologies, USA). The real-time PCR cycling condition is 40 cycles of 95°C 5 sec, 60°C 30 sec [17]. The sequences of the RT-PCR primers (RiboBio, China) were as follows (5ʹ – 3ʹ): LGALS3BP (forward: 5ʹ-AGGTACTTCTACTCCCGAAGGA-3ʹ, reverse: 5ʹ-GGCCACTGCATAGGCATACA-3ʹ) and GAPDH (forward: 5ʹ-GGT GAA GGT CGG TGT GAA CGG ATT T-3ʹ, reverse: 5ʹ-AAT GCC AAA GTT GTC ATG GAT GAC C-3ʹ). The relative mRNA expression was calculated using the 2^−ΔΔCt^ method and normalized to GAPDH expression.

### 2.7. Western blot analysis

Cells were lysed with RIPA lysis buffer (Beyotime Biotechnology, China) supplemented with PMSF inhibitor (Beyotime Biotechnology, China). 20 ug protein lysates were loaded and separated by 10% SDS-containing polyacrylamide gel (GenScript, China) and transferred to 0.22 μm polyvinylidene fluoride (PVDF) membranes (Bio-Rad, USA) in a condition of 120 V 60 min. The membranes were then blocked with Tris buffered saline Tween20 (TBST) containing 5% nonfat milk for 1 hour at room temperature, and incubated with primary antibodies overnight at 4°C. The membranes were then washed twice with TBST for 10 min each and incubated with HRP-linked secondary antibody for 1 hour, followed by washing three times in TBST for 10 min per wash. The bands were visualized using an enhanced chemiluminescence (ECL) kit (Thermo Fisher Scientific, USA) in Image quant LAS400 mini (GE Healthcare, Germany). Antibodies against the following proteins were used in the experiments: LGALS3BP (Abcam, England1:1000), Actin (Abcam, England 1:5000).

### 2.8. RNA sequencing analysis

The differentially expressed genes between MTX-resistant and original cell lines were identified by RNA-seq analysis (RiboBio, China), whose results were obtained by removing reads containing adapter, poly-N, and low-quality reads from the raw data. Next, the Q20, Q30, and GC contents of the clean data were calculated. Paired-end clean reads were aligned to the reference genome using STAR. Read numbers mapped to each gene were counted by HTSeq v0.6.0. Differential expression analysis of two conditions was performed using the DESeq^2^ package (1.10.1). The resulting P-values were adjusted using Benjamini and Hochberg approach for controlling the false discovery rate (FDR). Genes with an adjusted P_− value_ < 0.05, as identified by DESeq^2^, were considered to be differentially expressed.

### 2.9. Detection of LGALS3BP in choriocarcinoma samples by ELISA

Serum of patients with gestational trophoblastic neoplasia (GTN) which have different effects to MTX was collected at the Women’s Hospital, School of Medicine Zhejiang University between January 2016 and December 2019. The samples were classified according to drug resistance criteria [11] as either MTX-sensitive or MTX-resistant. All samples were collected as whole blood with EDTA anti-coagulation, then centrifugated at 3000 rpm for 10 min; the serum supernatant was transferred to new tubes and stored at −80°C for ELISA assays [18]. Sample collection was approved with the informed consent of each woman and by the institutional ethical review board of the hospital. The study was conducted according to the guidelines of the Declaration of Helsinki, and approved by the Institutional Review Board (or Ethics Committee) of Women’s Hospital, School of Medicine, Zhejiang University (protocol code: 2,015,068).

Twenty samples were classified as MTX-sensitive and twenty samples were classified as MTX-resistant. When samples preparation was completed, the concentration of LGALS3BP was determined with the Human Galectin-3 Binding Protein ELISA Kit (R&D, USA).

### 2.10. Plasmids construction and siRNA transfection

LGALS3BP and negative control plasmids were produced by GeneChem (RiboBio, China). Small-interfering RNAs (siRNAs) and negative controls were purchased from RiboBio. Transfections were performed using X-treme GENE HP DNA transfection reagent (Roche, USA) following the manufacturer’s instructions [19].

### 2.11. Statistical analysis

Statistical analysis was performed with the SPSS 20.0 software package (IBM, Armonk, NY). All experiments were repeated 3 times. All data are presented as the means ± SD. Shapiro-Wilk test was used to determine whether or not a sample comes from a normal distribution. The two tailed Student’s t test or one-way analysis of variance test was used to determine the significant differences. P < 0.05 was considered as statistically significant.

## 3. Results

This study aimed to analyze the effect of LGALS3BP on the effectiveness of MTX treatment in choriocarcinoma. After establishing two MTX-resistant choriocarcinoma cell lines by gradual dose escalation of MTX, we performed RNA-sequencing to explore the possible genes associated with MTX-resistance, and found LGALSBP was up-regulated in MTX-resistant choriocarcinoma cell lines in both RNA and protein level. Meanwhile, the sera concentration of LGALS3BP was higher in MTX-resistant patients than MTX-sensitive patients indicating LGALS3BP may be a molecular mark to predict MTX-resistance in choriocarcinoma. In order to further understand the function of LGALS3BP in MTX-resistance in choriocarcinoma, we overexpressed LGALS3BP in original cells and knocked down LGALS3BP in MTX-resistant cells and found overexpressing LGALS3BP can promote MTX-resistance, while knocking down LGALS3BP can reverse the MTX-resistance in choriocarcinoma.

### 3.1. Establishment of human choriocarcinoma cancer cells with acquired resistance to MTX

**Figure 1.**
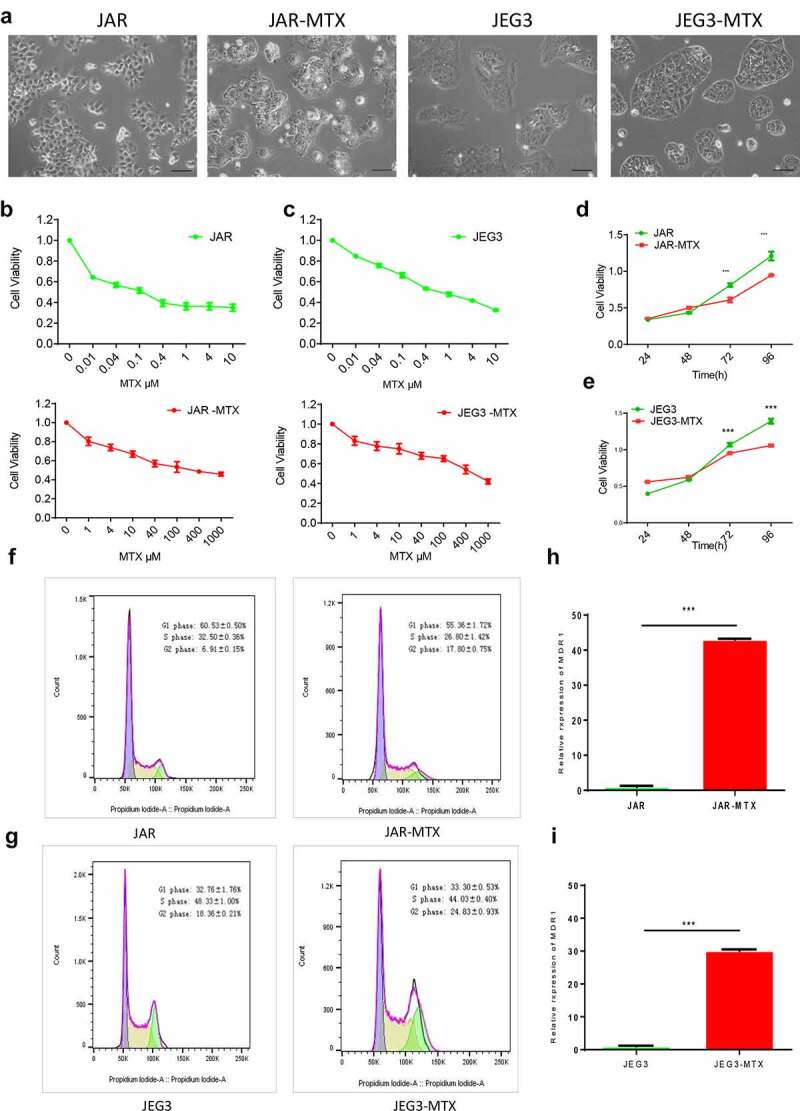
Evaluation of JAR and JEG3 cells with acquired resistance to MTX. (a) The cell morphology of MTX-resistant JAR, JEG3 and original cells under an inverted microscope. (b and c): The survival of JAR and JAR-MTX(b), JEG3 and JEG3-MTX(c) at various concentrations of methotrexate analyzed by CCK-8 assays. (d and e): Cell proliferation of JAR and JAR-MTX(d), JEG3 and JEG3-MTX(e) was determined by CCK-8 assay. F and G: Cell cycle of JAR and JAR-MTX(f), JEG3 and JEG3-MTX(g) was determined by flow cytometry analysis. H and I: The expression of multidrug resistance gene 1(MDR1) was detected by qRT-PCR in JAR and JAR-MTX(h), JEG3 and JEG3-MTX(i). Data are expressed as mean ± SD from at least three independent experiments. ** P < 0.01, *** P < 0.001.

We established a new cell line of human choriocarcinoma cancer cells with resistance to MTX. After an almost 1-year period of drug induction, we established MTX-resistant cells named as JAR-MTX, JEG3-MTX. The size or shape of JAR-MTX, JEG3-MTX are different from their original cells. To be detailed, there were more dark matter accumulation in the cells, especially near the capsule by an inverted microscope (Figure 1(a)). The IC_50_ of MTX in JAR-MTX, JEG3-MTX cells are significantly higher than their original cells (Figure 1(b, c)). The proliferation rate of MTX resistant cell is lower than that in its original cell analyzed by CCK-8 kit (Figure 1(d,e)), The cell cycle of both comparison pairs was analyzed by flow cytometry (Figure 1(f,g); Table 2). A prolonged G2-phase was found in MTX-resistant cells (JAR-MTX, JEG3-MTX) compared to their original cells (JAR, JEG3), indicating that after the introduction of drug MTX, the cells were DNA damaged and the G2-phase was blocked.

**Table 2.** The distribution of cell cycle in JAR and JAR-MTX, JEG3 and JEG3-MTX

	JAR	JAR-MTX	JEG3	JEG-MTX
G1 phase	60.5 ± 0.50%	55.3 ± 1.72%	32.7 ± 1.76%	33.3 ± 0.53%
S phase	32.5 ± 0.36%	26.8 ± 1.42%	48.3 ± 1.00%	44.0 ± 0.40%
G2 phase	6.91 ± 0.15%	17.8 ± 0.75%	18.3 ± 0.21%	27.8 ± 0.93%

The resistance of MTX-resistant cells to different chemotherapeutic agents was analyzed by cell viability assay. The results showed that MTX-resistant cells were resistant to MTX, as well as VP-16, 5-FU, TAXOL and ActD, the RI of MTX is 791.50 and 1040.04, concurrently it has cross resistance to VP-16, 5-FU, TAXOL and ActD, compared with JAR, JEG3 (Table 1). After drug-resistant cell lines were established, the cell lines were cultured to continue without drug about 3 months and half a year of liquid nitrogen cryopreservation, the RI to the MTX of drug resistance was detected. It turned out the respective stability was 93.2%and 90.1%, indicating the stability of MTX-resistant cells. The expression of multidrug resistance1 (MDR1) detected by qRT-PCR (Figure 1(d)), expression of MDR1 in drug-resistant cells is increased evidently, that was increased 43 times and 30 times compared to counterparts (P < 0.001). The results showed that MDR1 expression is related to the resistance of choriocarcinoma cell line, these findings formed the basis for further study of the resistance mechanism of choriocarcinoma.

**Table 1.** Relative resistance index of different chemotherapeutic drugs to the drug resistance of MTX cell lines of Choriocarcinoma cancer

MTX–resistant cell lines	Relative resistance index^a^				
	MTX	VP-16	5-FU	TAXOL	ACTD
JAR-MTX/JAR	791.5	2.31	4.45	2.47	2.74
JEG3-MTX/JEG3	1040.0	22.6	2.35	2.19	7.11

a: Relative resistance index is defined as the IC50 value of the drug resistance of MTX cells divided by the IC50 value of Choriocarcinoma cells.

### 3.2. Differentially expressed genes between MTX resistant cells and their original cells analyzed by RNA-seq

**Figure 2.**
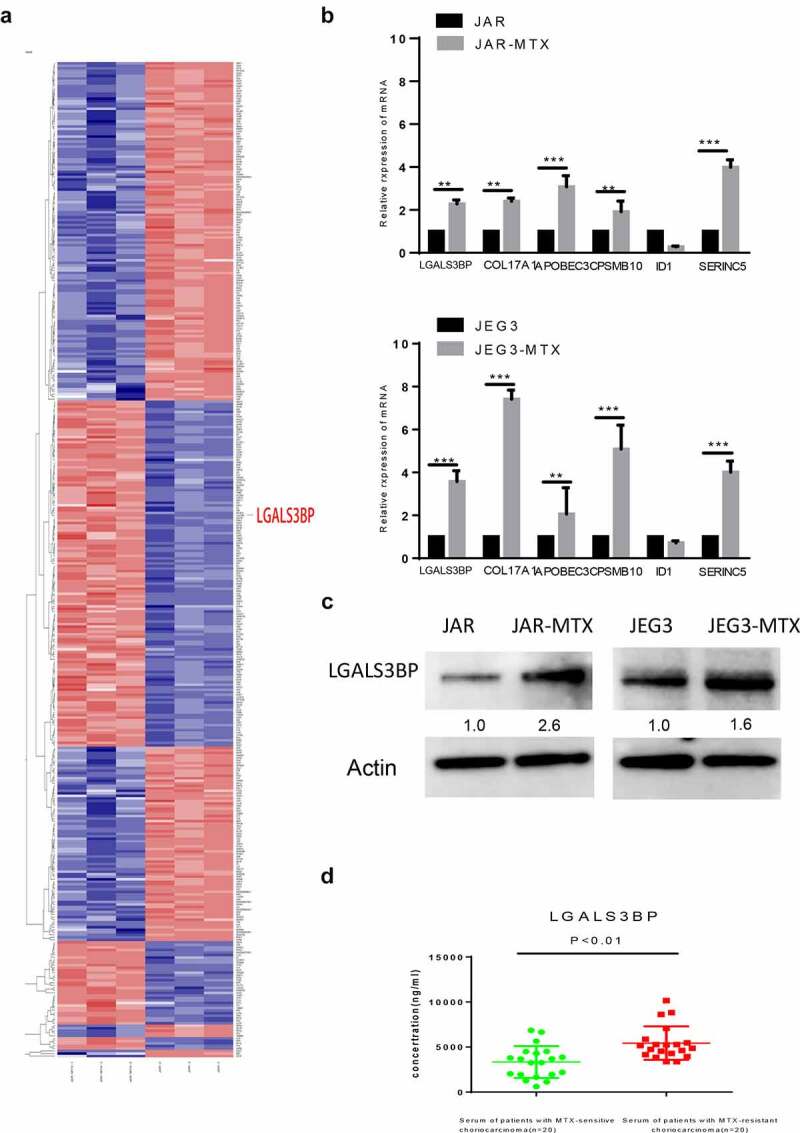
Verification of LGALS3BP overexpression in MTX-resistant GTN cells. (a) RNA-sequencing of differentially expressed genes in MTX-resistant and original GTN cells. (b) Determination of differentially expressed genes in MTX-resistant and original GTN cells by qRT-PCR. (c) Determination of LGALS3BP expression in MTX-resistant and original GTN cells by Western blot analysis. (d) Determination of LGALS3BP concentration in serum of patients with MTX-resistant choriocarcinoma compared with MTX-sensitive choriocarcinoma by ELISA. Data are expressed as mean ± SD of at least three independent experiments. ** P < 0.01, *** P < 0.001.

Total RNA was extracted from the paired-comparison groups JAR VS JAR-MTX and JEG3 VS JEG3-MTX.RNA-sequence (Ruibo, China) was used to screen for differentially expressed genes between MTX-resistant JAR, JEG3 cells and their counterparts. The heat map showed JAR-MTX VS JAR have 492 different genes, JEG3-MTX VS JEG3 have 220 different genes, that with significantly differential expression (fold change ≥2 or≤0.5) by DeCyderMS™2.0 analysis, as shown in Figure 2(a). There were 34 different genes in common, contain 11 up-regulated genes and 23 down-regulated genes in the MTX-resistant cells compare to their counterparts. After condition optimizing, we found six protein coding genes that were upregulated in drug-resistant cell lines.

### 3.3. Expression of LGALS3BP in MTX resistant cells and patient samples

After RNA-sequencing, we then explored the expression of six up-regulated genes was detected by qRT-PCR (Figure 2(b)). We focused on LGALS3BP, which was shown to be particularly up-regulated in MTX-resistant cells and had been proven to be an oncogene in several cancers, while its function in the regulation of MTX resistance in GTNs still remains poorly understood. The Western blot confirmed that LGALS3BP was overexpressed in MTX-Resistant cells (Figure 2(c)). Meanwhile, we explored the concentrations of LGALS3BP in patient serum with an ELISA Kit. The concentration of LGALS3BP in patient serum of MTX-sensitive choriocarcinoma was 621.39 ~ 6651.35 ng/ml, and the concentration of LGALS3BP in patient serum of MTX-resistant choriocarcinoma was3376.00 ~ 10,157.90 ng/ml. The concentration of LGALS3BP was significantly elevated in serum samples from patients with MTX-resistant choriocarcinoma compare to MTX-sensitive choriocarcinoma (P < 0.01) (Figure 2(d); Table 3). These findings suggest that LGALS3BP plays a role in drug resistance to MTX in choriocarcinoma cancer.

**Table 3.** The LGALS3BP concentration in serum of patients with MTX-resistant choriocarcinoma compared to that in MTX-sensitive choriocarcinoma

	LGALS3BP concentration(ng/ml)
Serum of patients with MTX-sensitive choriocarcinoma(n = 20)	621.39–6651.35
Serum of patients with MTX-resistant choriocarcinoma(n = 20)	3376.00–10,157.90

### 3.4. Regulation of LGALS3BP and changes in drug resistance of MTX

**Figure 3.**
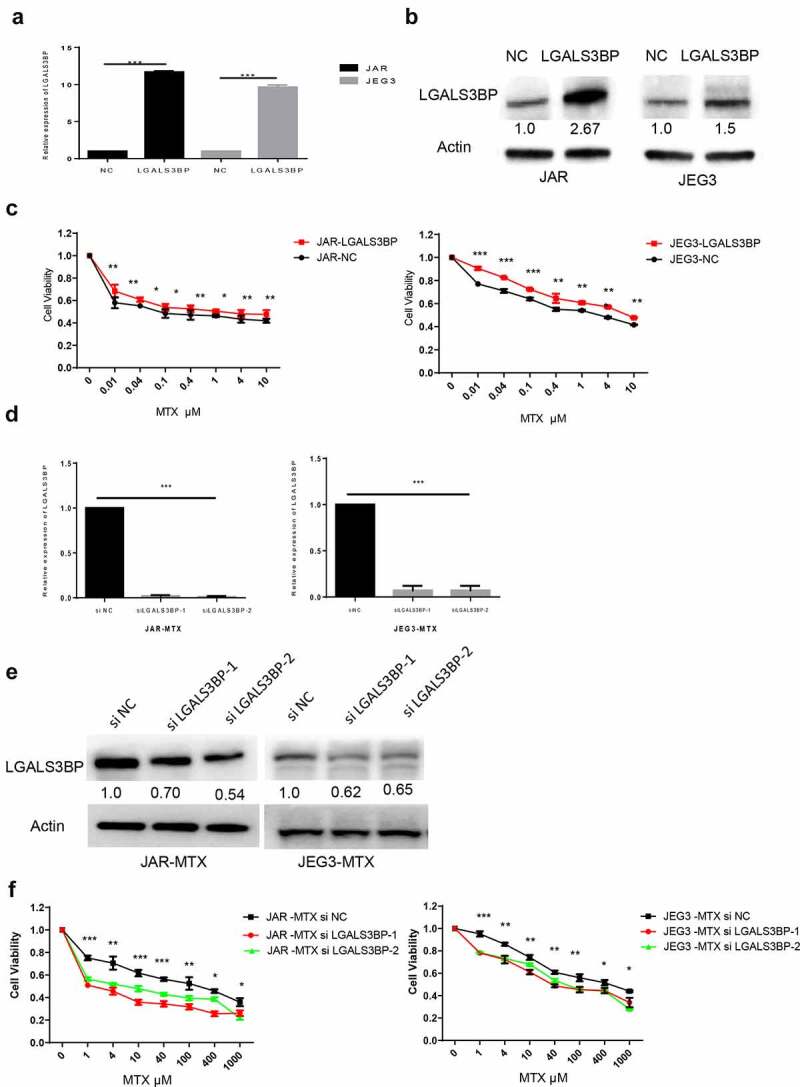
LGALS3BP promotes MTX resistance in JAR and JEG3 cells. (a) Determination of LGALS3BP expression in LGALS3BP-overexpressed JAR and JEG3 cells by qRT-PCR. (b) Determination of LGALS3BP expression in LGALS3BP-overexpressed JAR and JEG3 cells by Western blot analysis. (c) Determination of IC_50_ in LGALS3BP-overexpressed JAR and JEG3 cells by cell viability assay. (d) Determination of LGALS3BP expression in LGALS3BP- knockdown JAR-MTX and JEG3-MTX cells by qRT-PCR. (e) Determination of LGALS3BP expression in LGALS3BP-knockdown JAR-MTX and JEG3-MTX cells by Western blot analysis. (f) Determination of IC_50_ in LGALS3BP-knockdown JAR-MTX and JEG3-MTX cells by cell viability assay. Data expressed as mean ± SD from at least three independent experiments. *P < 0.05, ** P < 0.01, *** P < 0.001.

To determine the relationship between resistance and expression of LGALS3BP, LGALS3BP expression was over-expressed by plasmid vector and silenced by siRNA (siLGALS3BP). Choriocarcinoma cancer cells were transfected with plasmid for 48 H; expression of LGALS3BP was detected by q-PCR and Western blot (Figure 3(a, b)), which show that expression of LGALS3BP was increased. Plasmid transfected for 48 H, choriocarcinoma cancer cells were treated with 0.01, 0.04, 0.1, 0.4, 1, 4 and 10 μM MTX. Results showed that LGALS3BP overexpressed cells showed higher IC_50_ compared to control cells (Figure 3(c)). MTX-resistant cells were transfected with siRNA (siLGALS3BP) for 48 H; expression of LGALS3BP was detected by q-PCR and Western blot (Figure 3(d, e)), which show that expression of LGALS3BP was decreased. SiRNA (siLGALS3BP) transfected for 48 H, MTX-resistant cells still treated with MTX, that concentrations were 1, 4, 10, 40, 100, 400, 1000 μM. Results showed that the siRNA (siLGALS3BP)-transfected drug resistance of MTX cell lines showed lower IC_50_ compared to the drug resistance cells (figure 3(f)). These findings show that choriocarcinoma cancer cells with over-expressed LGALS3BP have increased resistance to MTX. In contrast, MTX-resistant cell lines with silenced LGALS3BP (siRNA: siLGALS3BP) showed decreased resistance to MTX.

## 4. Discussion

Choriocarcinoma characterized by human chorionic gonadotropin hormone elevation, is one of the most aggressive gestational trophoblastic neoplasias (GTN). Although it is rare in western country, 1 in 2882 pregnant women will develop choriocarcinoma in China. Although chemotherapy especially MTX treatment has been considered as the first-line therapy in treating choriocarcinoma due to the high sensitivity of choriocarcinoma to chemotherapy, a part of patients may fail to achieve complete remission after single-agent MTX treatment because of MTX resistance in clinical practice. Meanwhile, the mechanism of MTX resistance in choriocarcinoma still remains poorly understood. It is reported that activation of interferon signaling, inhibition of cell death/apoptosis, involvement of cancer stem cells, dysregulation of long non-coding RNAs, up-regulation of drug target gene and drug efflux may all contribute to the drug resistance in choriocarcinoma [7,20–23]. Considering that MTX treatment is not only a standard treatment for multiple kinds of cancers such as leukemia, breast cancer, but also wildly used in autoimmune diseases [24], unveiling the mechanisms involved in MTX resistance is of great significance. In order to explore the mechanisms involved in MTX resistance in choriocarcinoma, we successfully established two MTX-resistant cells by gradual dose escalation of MTX.

LGALS3BP was initially identified as a heavily glycosylated protein in tumor tissue and serum from breast cancer patients and lung cancer cell lines [25,26]. It is reported that elevated expression of LGALS3BP in either serum or tumor tissue is associated with poor clinical outcomes in patients with breast cancer, lung cancer, pancreatic carcinoma, hepatocellular carcinoma, pleural mesothelioma, and neuroblastoma [19]. However, the function of LGALS3BP in drug resistance and GTN is unclear. In this study, we found that LGALS3BP is overexpressed not only in MTX-resistant JAR and JEG3 cells but also in serum samples from patients with MTX-resistant choriocarcinoma. Meanwhile, LGALS3BP plays an important role in regulating drug resistance in GTN cells, as knockdown of LGALS3BP can attenuate drug resistance in MTX-resistant JAR and JEG3 cells while over-expression of LGALS3BP aggravates drug resistance in MTX-resistant JAR and JEG3 cells. However, we didn’t perform further research to better know how LGALS3BP mediates MTX resistance. Considering that LGALS3BP can augmented PI3K/Akt and ERK signaling pathways [12] and p-AKT, p-ERK1/2, p-STAT3 levels are markedly higher in MTX-resistant choriocarcinoma cells than in parental MTX-sensitive cell [7], it is reasonable to speculate that LGALS3BP may promote MTX resistance by PI3K/Akt and ERK signaling pathways. LGALS3BP is a secreted protein that can interact with several members of the extracellular matrix such as integrin, fibronectins, galectins, laminins, and tetraspanins, LGALS3BP may also contribute to MTX resistance directly by affecting drug efflux. Further research is needed to determine the resistance mechanisms of LGALS3BP in GTN to draw a clearer conclusion, and to explore the clinical potential of targeting LGALS3BP to sensitize GTN cells to chemotherapeutic drugs.

As we can see in Table 1, the MTX-resistant cells were not only resistant to MTX, but also resistant VP-16, 5-FU, TAXOL and ACTD. It is reasonable to speculate that LGALS3BP may also play important role in VP-16, 5-FU, TAXOL and ACTD, which needs further study.

In summary, we preliminarily established two MTX-resistant cells, and performed RNA-seq, and found LGALS3BP may play important role in MTX-resistance. We also used siRNA and plasmids to regulate the expression of LGALS3BP and found LGALS3B can regulate MTX sensitivity. Our work not only provides a research tool (MTX-resistant cells) for other researchers, but gives some hint on how MTX resistance is regulated.

## 5. Conclusions

Our data indicate that LGALS3BP contributes to MTX-resistance in choriocarcinoma. Our findings suggest the potential of LGALS3BP as a useful prognostic marker and a promising therapeutic strategy for development of more effective choriocarcinoma therapies.

## Highlights

1. MTX-resistant choriocarcinoma cell lines were induced by gradual dose escalation.

2. LGALS3BP was up-regulated in MTX-resistant cells compared to original cells.

3. MTX-resistant patients had higher sera concentration of LGALS3BP.

4. LGALS3BP promotes MTX resistance in choriocarcinoma cell lines
